# Dynamic changes in bronchoalveolar macrophages and cytokines during infection of pigs with a highly or low pathogenic genotype 1 PRRSV strain

**DOI:** 10.1186/s13567-017-0420-y

**Published:** 2017-02-27

**Authors:** Patricia Renson, Nicolas Rose, Mireille Le Dimna, Sophie Mahé, André Keranflec’h, Frédéric Paboeuf, Catherine Belloc, Marie-Frédérique Le Potier, Olivier Bourry

**Affiliations:** 1 Anses, Laboratoire de Ploufragan-Plouzané, Unité Virologie et Immunologie Porcines, Zoopôle, BP53, 22440 Ploufragan, France; 2 Anses, Laboratoire de Ploufragan-Plouzané, Unité Epidémiologie et Bien-Etre du Porc, Zoopôle, BP53, 22440 Ploufragan, France; 3 Anses, Laboratoire de Ploufragan-Plouzané, Service Production de Porcs Assainis et d’Expérimentations,, Zoopôle, BP53, 22440 Ploufragan, France; 4 Université Bretagne Loire, Rennes, France; 5 BIOEPAR, Oniris, INRA, 44307 Nantes, France; 6 UGPVB, 104 rue Eugène Pottier, CS 26553, 35065 Rennes, France

## Abstract

Porcine reproductive and respiratory syndrome virus (PRRSV) replicates primarily in pulmonary alveolar macrophages (PAMs) and the resulting lung damage is influenced by strain virulence. To better understand the pathogenesis of PRRSV infection, we performed a longitudinal study of the PAM population and lung cytokines in specific pathogen-free pigs infected either with the highly pathogenic Lena strain or with the low pathogenic Finistere strain in comparison to uninfected pigs. Bronchoalveolar lavage fluid (BALF) and blood were collected to follow viral, cellular and cytokine changes in lung with respect to clinical signs and systemic events. Compared to Finistere-infected pigs, Lena-infected pigs exhibited more severe clinical signs and 10- to 100-fold higher viral loads in BALF and blood. Similarly, they showed an earlier drop in BALF cell viability and phagocytic activity along with a decrease in the macrophage count. From 8 to 15 days post-infection (dpi), monocytes increased both in BALF and blood from Lena-infected pigs. BALF and blood showed contrasting cytokine patterns, with low increase of IFN-α and TNF-α levels and high increase for IL-1α and IL-8 in BALF after Lena-infection. In contrast, in the blood, the increase was marked for IFN-α and TNF-α but limited for IL-1β and IL-8. Down-regulation of PAM functions combined with inflammatory cytokine and monocyte recruitment may promote lung pathogenesis and virus replication in PRRSV infections with the highly pathogenic Lena strain. In contrast, the low pathogenic Finistere strain showed prolonged viral replication in lung, possibly related to the weak IFN-γ response.

**Electronic supplementary material:**

The online version of this article (doi:10.1186/s13567-017-0420-y) contains supplementary material, which is available to authorized users.

## Introduction

Porcine reproductive and respiratory syndrome (PRRS) is one of the most costly diseases for the swine industry worldwide, affecting both breeding and growing pigs [1]. PRRS is a viral disease that is characterized by reproductive failure in sows and by respiratory disorders, growth retardation and increased mortality in young piglets. The clinical manifestation of the disease depends on the PRRS viral strain, host and environmental factors and the presence of other pathogens [2].

The causative agent of PRRS is an enveloped virus with a 15 kb positive polarity single-stranded RNA genome, a member of the *Arteriviridae* family [3]. Strains of the PRRS virus (PRRSV) cluster into two different genotypes, initially geographically separated: the European genotype 1 and the North American genotype 2. Currently, both genotypes circulate globally [4]. PRRSV strains show great genetic and antigenic diversity along with large differences in pathogenicity [5]. PRRSV strains that have emerged in Eastern Europe in the past decade have widely extended the diversity of genotype 1 which is now divided into four subtypes [6]. Strains identified as genotype 1 subtype 3 (1.3) show higher pathogenicity in pigs than genotype 1.1 strains that have historically circulated in Europe, inducing high fever and severe respiratory disorders that exacerbate morbidity and mortality [7, 8]. The introduction of subtype 3 in Western Europe has the potential to adversely affect the large swine industry found in these countries.

After oronasal transmission, PRRSV initially colonizes the respiratory tract and plays an immunomodulatory role that delays or weakens host responses, leading to long virus persistence in the host [9]. The main target cells for virus replication are pulmonary alveolar macrophages (PAMs) [10]. Macrophages play a central role in immunity interfacing innate and adaptive responses. In lung, PAMs are crucial cells in homeostasis and defence against microbial invasion with particular ability to recognize and clear pathogens without inducing inflammation that disturbs gas exchanges [11]. These cells are highly phagocytic and express numerous receptors to recognize pathogens and initiate the innate immune response [12]. In haemostatic conditions, PAMs represent the major cell type found in the alveolar environment and low cytokine levels are measured. After pulmonary infection or injury that overwhelms this first-line defence mechanism, cellular and molecular changes can occur toward a strong inflammatory process. PAMs can produce a large range of cytokines and chemokines that mediate the activation or the recruitment of various immune cells into the lungs to reinforce host defences against pathogen invasion [12–14].

Pro-inflammatory cytokines, such as interferon-alpha (IFN-α), tumour necrosis factor-alpha (TNF-α) and interleukin (IL)-1 have been shown to play a key role in respiratory disease severity. Compared to other respiratory pathogens, PRRSV infection induces limited pro-inflammatory cytokine production in the lungs [15]. However, in pigs infected by highly pathogenic (HP) strains, high levels of TNF-α, IL-1 and IL-6 have been quantified in lung or in blood [16–19].

To better understand the pathogenicity of HP-PRRSV strains, especially at the pulmonary level, we compared the dynamic changes induced in alveolar environment after the genotype 1.3 HP Lena strain infection with those induced by the genotype 1.1 low pathogenic (LP) Finistere strain infection. Previous papers on Lena strain pathogenicity mainly focused on blood by longitudinal sample collection [17] or in the lungs but only through sequential slaughtering [20]. Our study presents an original strategy combining simultaneous local and systemic analyses to identify lung-specific modulations in cellular populations, viability and phagocytic activity, as well as in cytokine levels. We focused our study on PAMs using a longitudinal monitoring for 42 days post-infection (dpi) to detect any correlations between clinical, virological, and immunological parameters. Moreover, we used specific pathogen-free (SPF) pigs with very high health level, thereby avoiding any interference with other respiratory pathogens.

## Materials and methods

### Viruses

The LP genotype 1.1 Finistere PRRSV strain (PRRS-FR-2005-29-24-1) was isolated in France in 2005 from a herd with reproductive failures in sows (abortions). In SPF pigs, Finistere infection induces a mild clinical expression [21]. The HP genotype 1.3 Lena PRRSV strain was kindly provided by Dr Hans Nauwynck, (University of Ghent, Belgium). The Lena strain was isolated in Belarus in 2007 from a herd with mortality, reproductive failures and respiratory disorders [7]. Both viruses were propagated and titrated on PAMs for 2 and 5 passages for the Finistere and the Lena strains, respectively.

### Animal experiment design

Six-week-old SPF Large White pigs from our biosecurity level-3 air-filtered facilities, with high health status and, in particular, free of numerous respiratory pathogens such as PRRSV, swine Influenza virus, porcine circovirus type 2, *Mycoplasma hyopneumoniae*, *Actinobacillus pleuropneumoniae*, *Pasteurella multocida*, *Bordetella bronchiseptica* and *Haemophilus parasuis*, were randomly assigned to three groups housed in three separate rooms: one group of eight animals (Lena group) and two groups of five animals (Finistere group and Control group). Pigs were inoculated by nasal route with either the Finistere strain or Lena strain (5.10^5^ CPD_50_/pig): 2.5 mL of the viral suspension was injected into each nostril using a 5 mL syringe without a needle. The Control group was mock-inoculated with MEM culture media.

Rectal temperature, weight gain and clinical score were monitored daily using a template from Weesendorp et al. [17]. Hyperthermia was reported for rectal temperature higher than 40 °C. Blood samples and bronchoalveolar lavage fluid (BALF) were collected before infection (on day-3) and then at 2, 4, 8, 11, 15, 22, 30, 36 and 43 dpi. BALF collections were obtained by infusing 2 × 20 mL of warm, sterile PBS instilled in the lungs through a nasogastric probe of 4.5 mm of diameter and 50 or 57 cm long depending on the age and the weight of the animals (Laboratoires Euromedis, Neuilly-sous-Clermont, France). The BALF collection was performed under general anaesthesia obtained by intravenous injection of 5 mg/kg ketamine (Clorketam 1000, Vétoquinol SA, Lure, France) and 1 mg/kg xylazine (2% Rompun, Bayer HealthCare, Leverkusen, Germany). The mean BALF volume collected using this procedure was 25 mL. The rare BALF with blood contamination were excluded from all the analyses. All pigs were euthanised at 44 or 45 dpi. Post-mortem examination was conducted on all pigs after euthanasia.

The animal experiment was authorised by the French Ministry for Research (Authorisation no. 00676.01) and approved by the national ethics committee (authorisation no. 09/07/13-1).

### Sample treatment

Serum samples were purified from coagulated blood samples by centrifugation at 3000 ×* g* for 5 min. From EDTA blood samples, blood cell counts were obtained using a MS9.5 haematology analyser (Melet Schloesing Laboratoires, Osny, France).

Cells were purified from BALF by centrifugation at 500 × *g* for 10 min at 4 °C and counted using a haemocytometer with Trypan blue staining to determine the percentage of viability. BALF cells were monitored by flow cytometry for phenotypic changes and phagocytic activity. The remaining cell-free BALF were frozen at −70 °C.

### Viral genome quantification

RNA was purified from serum or cell-free BALF using the NucleoSpin RNA 8 virus (Macherey-Nagel, Düren, Germany) according to the manufacturer’s instructions. Specific genome detection for either the Lena strain or the Finistere strain was assessed by qRT-PCR using the SuperScript III Platinum one step qRT-PCR kit (Life Technologies, Carlsbad, CA, USA) using specific primers and probes of the target gene (Finistere strain ORF5: forward primer TATGCGAGCTGAATGGGACC, reverse primer AGGATATGAGTGGCAACCGG, probe 6FAM-TGGGCAGTTGAGACTTTCGTGCT-TAM or Lena strain ORF7: forward primer AGAACCAGCGCCAATTCAGA, reverse primer TCTTTTTCGCCTGTCCTCCC, probe 6FAM-AAACACAGCTCCAATGGGGAATGGC-TAM) or of the internal control (porcine beta-actin: forward primer CTCGATCATGAAGTGCGACGT, reverse primer GTGATCTCCTTCTGCATCCTGTC, probe TET-ATCAGGAAGGACCTCTACGCCAACACGG-BHQ1). RT-PCR reactions were performed with the Chromo4 real-time PCR device (Bio-Rad, Hercules, CA, USA) for 30 min at 50 °C, 2 min at 94 °C followed by 45 cycles of 15 s at 94 °C and 30 s at 60 °C. Genome quantifications were obtained using serial dilutions of Finistere or Lena strain (known infectious titres) in serum or cell-free BALF collected from SPF pigs. Results are given in equivalent (eq) CPD_50_/mL of serum or BALF.

### Antibody assessment

Antibodies against PRRSV were detected using IDEXX PRRS X3 ELISA tests (IDEXX laboratories, Liebefeld, Switzerland) according the manufacturer’s instructions in serum or using a protocol modified for BALF (starting dilution at 1:2 instead of 1:40).

### Flow cytometry cellular phenotype and phagocytosis analyses

BALF cells (5.10^5^ cells) were transferred to microtiter plates and washed with PBS for 3 min at 350 × *g*. For phenotype analyses, cells were single- or triple-stained for swine membrane markers [22], using the following primary mouse monoclonal antibodies: phycoerythrin (PE)-conjugated anti-porcine CD21 (clone BB6-11C9.6), fluorescein isothiocyanate (FITC)-conjugated anti-porcine CD3e (clone PPT3), PE-conjugated anti-SWC3 (also identified as CD172a; clone 74-22-15), FITC-conjugated anti-SWC9 (also identified as CD203a; clone PM18-7), unconjugated anti-SWC8 (clone MIL3), or stained with the appropriate mouse isotype control: FITC or PE-conjugated IgG1 (clone MOPC-21) (all obtained from Bio-Rad, Hercules, CA, USA, SouthernBiotech, Birmingham, AL, USA or Becton, Dickinson and Company, Franklin Lakes, NJ, USA). The unlabelled primary antibody was detected by goat polyclonal secondary antibody allophycocyanin (APC)-conjugated anti-mouse IgM (Abcam, Cambridge, UK) which was also used as isotype control. Same cell populations were identified in BALF by flow cytometry as in blood using the haematology analyser to compare population changes between the two locations. For phagocytosis tests, cells were incubated with TransFluoSpheres 488/560 nm fluorescent microspheres (10 microspheres per cell) for 1 h at 37 °C (Life Technologies, Carlsbad, CA, USA) as described by De Baere et al. [23]. For phenotyping and phagocytosis tests, flow cytometry analyses were performed on a constant event number of 10 000 cells fixed with 4% paraformaldehyde, acquired on a FC500 flow cytometer using MXP 2.1 and analysed using Kaluza 1.2 software (all from Beckman Coulter, Fullerton, CA, USA). Cytometer parameters and gating were defined in order to remove cell debris and free microspheres from the acquired events. Phenotype data were obtained by analysing marker expression on the cell surface in the appropriate gate, as described by Nielsen et al. on SSC and FSC parameters for lymphocytic and monocytic cells or for macrophages [24]. For phagocytosis analysis, the median fluorescence intensity (MFI) was measured, reflecting the quantity of microspheres captured per cell.

### Cytokine measurements

Porcine cytokines were quantified in serum and cell-free BALF using ELISA kits from Bio-techne for TNF-α, IL-1β, IL-8, IL-10 and IFN-γ (Bio-techne, Minneapolis, MN, USA). Porcine IFN-α was detected using an in-house ELISA test, as previously described [25].

Porcine IL-10 and IFN-γ gene expressions were assessed in cells isolated from BALF using the TaqMan-based triplex real-time RT-PCR described by Petrov et al. which uses both β-actin and GAPDH as endogenous controls to normalise the cytokine gene expression [26]. Petrov’s RT-PCR was performed with modified protocol using the SuperScript III Platinum One-Step Quantitative RT-PCR System (Life Technologies, Carlsbad, USA) and previously published primers and probes for IL-10 and IFN-γ cytokines [27]. The relative expressions of target genes were calculated using the R = 2^−∆∆CT^ equation [28] with regard to the expression level in BALF cells of each pig before the infection (−3 dpi).

### Statistical tests

The measured variations of each parameter for an infected group were compared with that of the Control group or the other infected group and analysed daily using a global Kruskal–Wallis test to detect global significant differences between groups. Then, a Holm’s adjusted pairwise Wilcoxon-Mann-Whitney test was added to identify specific significant differences between groups. Correlation analyses were performed using Spearman’s matrix. Statistical tests were performed using R software (R Development Core Team, 2008), with the limit of significance set at *p* < 0.05. The error bars on the graphs represent the standard error (SE).

## Results

### Clinical data

**Figure 1:**
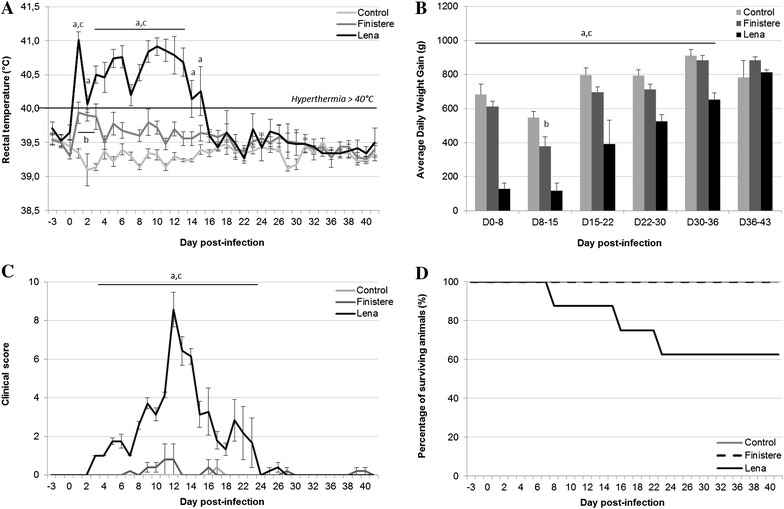
**Clinical parameters. A** Rectal temperature, **B** average daily weight gain, **C** clinical score and **D** survival were monitored. All data are reported as the mean (±SE) of results obtained from pigs (*n* = 5) in the Control or Finistere group or the surviving pigs (*n* = 5–8) in the Lena group. “a” indicates a significant difference between the Control and Lena groups, “b” a significant difference between the Control and Finistere groups and “c” a significant difference between the Lena and Finistere groups using a global Kruskal–Wallis test followed by a Holm’s adjusted pairwise Wilcoxon-Mann-Whitney test (*p* < 0.05).

Animals inoculated with the Finistere strain showed mild signs of the disease with an increase in rectal temperature below the hyperthermic threshold and a very low clinical score (Figures 1A and C). Only minor respiratory troubles (mild dyspnea, some sneezing and cough) and nasal discharge were observed for pigs infected by the Finistere strain. One pig also showed typical “blue ears” at 11–12 dpi. Weight monitoring of Finistere-infected pigs revealed a significant reduction in the average daily weight gain (ADWG) the second week of infection (Figure 1B). All pigs recovered from the infection at the end of the experiment at 44–45 dpi. No characteristic lesions were observed during necropsy, except for tracheobronchial, mediastinal, inguinal and iliac reactive lymph nodes.

In contrast, pigs infected with the Lena strain showed a long period of hyperthermia from 1 to 15 dpi with a marked clinical score from 3 to 22 dpi that peaked at 12 dpi (Figures 1A and C). Severe respiratory disorders with high breathing frequency, cough, sneezing and nasal discharge were observed. Apathy and anorexia were also recorded and the ADWG of the Lena group dropped significantly from the first (*p* = 0.007) to the fifth (*p* = 0.035) week of infection compared with either the Control group or the Finistere group (Figure 1B).

During the experiment, 3 of the 8 pigs died in the Lena group (Figure 1D): animal #4915 was found dead at 8 dpi whereas its symptoms were not high enough to be sacrificed the day before. Pigs #4917 and #4853 had to be euthanised respectively at 16 dpi due to very high clinical score and at 22 dpi due to severe anaemia. Necropsy of pigs #4915 and #4917 revealed thymus atrophy, diaphragmatic rupture, pleurisy, splenomegaly, fibrinous peritonitis, as well as hypertrophied and haemorrhagic lymph nodes. Pig #4853 showed thymus atrophy, pneumonia lung lesions, hypertrophied lymph nodes and blood in the stomach and large intestine. As observed in Finistere-infected pigs, no specific lesions except for reactive lymph nodes were found during necropsy of Lena-infected pigs that had recovered from the disease.

### Haematological data

**Figure 2:**
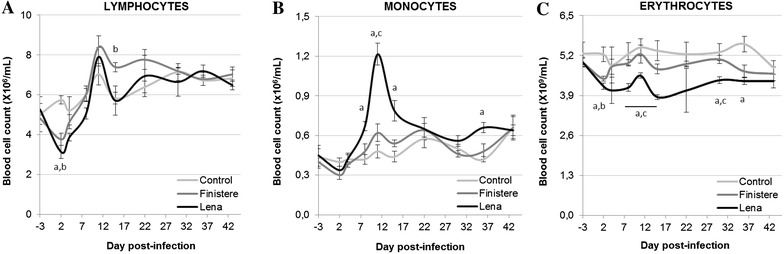
**Blood cell count. A** Monocyte, **B** lymphocyte and **C** erythrocyte counts were obtained using an haematology analyser. All data are reported as the mean (±SE) of results obtained for the pigs (*n* = 5) in the Control or Finistere groups or the surviving pigs (*n* = 5–8) in Lena group. “a” indicates a significant difference between the Control and Lena groups, “b” a significant difference between the Control and Finistere groups and “c” a significant difference between the Lena and Finistere groups using a global Kruskal–Wallis test followed by a Holm’s adjusted pairwise Wilcoxon-Mann-Whitney test (*p* < 0.05).

Blood lymphocyte counts rapidly decreased in PRRSV-infected pigs (at 2 dpi) without any significant difference between strains. Thereafter, the lymphocyte population reached the Control group levels at 11 dpi and then exceeded them in the Finistere-infected group at 14 dpi (Figure 2A). A four-fold higher blood monocyte count was detected from 8 to 14 dpi in the Lena group, whereas the increased monocyte level observed at 11 and 14 dpi was not significant in the Finistere group (Figure 2B). Similar cell dynamics were observed for blood granulocytes, with a significant increase in the granulocyte count found at 11 dpi in the Finistere group compared with the Control group, but still lower than those observed in the Lena group (data not shown). Decreased erythrocyte counts were observed for both PRRSV strains at 2 dpi, however the duration and the intensity of the decrease was exacerbated in the Lena group (Figure 2C). Similar patterns were observed for haemoglobin levels (data not shown).

### Viral load and antibody levels in serum and BALF

**Figure 3:**
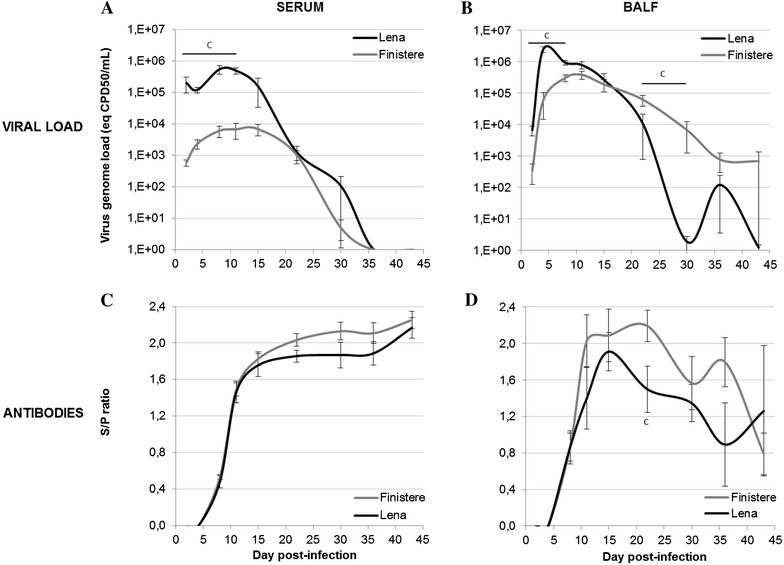
**PRRSV viral load and antibodies in serum and BALF from infected groups.** PRRSV genome load was quantified (**A**) in serum and (**B**) in BALF by RT-PCR. Antibodies against PRRSV N protein were detected (**C**) in serum and (**D**) in BALF using ELISA with a positive threshold set at 0.4. All data are reported as the mean (±SE) of results obtained for the pigs (*n* = 5) in the Finistere group or the surviving pigs (*n* = 5–8) in Lena group. “c” indicates a significant difference between the Lena and Finistere groups using a global Kruskal–Wallis test followed by a Holm’s adjusted pairwise Wilcoxon-Mann-Whitney test (*p* < 0.05).

Both strains were detected by RT-PCR as early as 2 dpi in the sera of all infected animals. The Lena strain viral load was 100-fold higher than those of the Finistere strain until 11 dpi (Figure 3A). Viral load of both PRRSV strains decreased at 21 dpi and were not detected from 36 dpi in sera. In BALF, similar viral dynamics were observed (maximum mean viral load of 2.5.10^6^ eq CPD_50_/mL at 4 dpi for Lena vs 3.8.10^5^ eq CPD_50_/mL at 11 dpi for Finistere), except that the rates of decrease of the Finistere strain viral load was significantly slower than those of the Lena strain from 22 dpi (*p* = 0.017 and 0.022 at 22 and 30 dpi, respectively) (Figure 3B). At 30 dpi, the virus was only detected in BALF of one out of five Lena-infected pigs, whereas the five Finistere-infected pigs were still PRRSV positive.

PRRSV-specific antibodies were detected using ELISA from 8 dpi in serum and BALF collected from Lena- or Finistere-infected animals, with slightly lower S/P values for the Lena strain (Figures 3C and D).

### Cell viability and phagocytic activity in BALF

**Figure 4:**
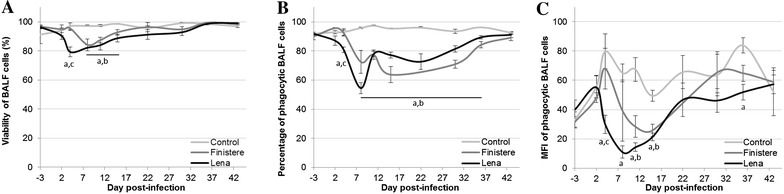
**BALF cell viability and phagocytic activity. A** Viability of BALF cells was evaluated using haemocytometer with Trypan blue. **B** Percentage and **C** median of fluorescence intensity of phagocytic BALF cells were assessed by flow cytometry. All data are reported as the mean (±SE) of results obtained for the pigs in Control or Finistere group (*n* = 5 for each) or the surviving pigs (*n* = 5–8) in Lena group. “a” indicates a significant difference between the Control and Lena groups, “b” a significant difference between the Control and Finistere groups and “c” a significant difference between the Lena and Finistere groups using a global Kruskal–Wallis test followed by a Holm’s adjusted pairwise Wilcoxon-Mann-Whitney test (*p* < 0.05).

Cell counts showed an increase of cellular concentration in BALF with the age of the animals for the 3 groups but with no difference between groups (data shown in Additional file 1). For both strains, the viability of BALF cells decreased during the first two weeks after infection (Figure 4A). The proportion of phagocytic cells in BALF was also altered by PRRSV infection from 8 to 36 dpi (Figure 4B). Furthermore, the MFI measurement, reflecting the quantity of phagocytosed beads per cell, demonstrated a reduction in phagocytic activity in BALF cells after PRRSV infection (Figure 4C). For the Lena strain, the decrease in the viable or phagocytic BALF cell count, as well as the reduction of their phagocytic activity, was observed as early as 4 dpi and reached minimum values lower than those for the Finistere strain (79.3 vs. 84.3%, 54.5 vs. 63.8% and 11.0 vs. 25.9 for cell viability, phagocytic cells percentage and MFI of phagocytic BALF cells isolated from Lena- vs. Finistere-infected pigs, respectively). Numerous cell debris were observed among BALF cells and particularly at 8 dpi in Lena-infected group and at 11 dpi in Finistere-infected pigs.

BALF cell viability was inversely correlated with viral load in BALF calculated for the entire duration of the experiment (r = −0.72 and −0.57 for Lena- and Finistere-infected group respectively). A similar correlation was observed between phagocytic BALF cell percentage and viral load in BALF (r = −0.61 for the Lena- or Finistere-infected group).

### Dynamics of cell subsets in BALF

**Figure 5:**
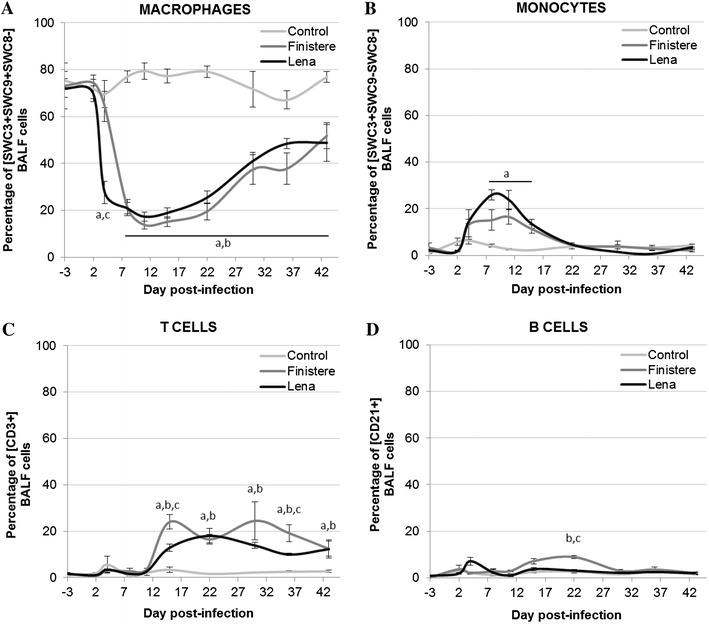
**BALF cell subsets.** Percentages of (**A**) [SWC3+ ,SWC9+ , SWC8−] macrophages, (**B**) [SWC3+ ,SWC9−, SWC8−] monocytes, (**C**) [CD3+] T cells and (**D**) [CD21+] B cells were measured in BALF using flow cytometry. All data are reported as the mean (±SE) of results obtained for the pigs in the Control or Finistere groups (*n* = 5 in each group) or the surviving pigs (*n* = 5–8) in the Lena group. “a” indicates a significant difference between the Control and Lena groups, “b” a significant difference between the Control and Finistere groups and “c” a significant difference between the Lena and Finistere groups using a global Kruskal–Wallis test followed by a Holm’s adjusted pairwise Wilcoxon-Mann-Whitney test (*p* < 0.05).

Without infection, [SWC3+ , SWC9+ , SWC8−] macrophages constituted the main population within BALF cells. After PRRSV infection, the proportion of macrophages decreased from 4 dpi with the Lena strain and from 8 dpi for the Finistere strain (Figure 5A). Thereafter, the percentage of macrophages remained below 60% until the end of the experiment without any differences between strains. Correlation analyses showed that, for both strains, BALF macrophage count was associated with phagocytic cell percentage (r = 0.72 and 0.86 for Lena and Finistere, respectively) and was inversely related to viral load (r = −0.63 and −0.74 for Lena and Finistere, respectively).

Conversely, the [SWC3+ , SWC9−, SWC8−] monocyte population increased in BALF from 8 to 15 dpi (up to 26% for Lena, 17% for Finistere), the increase being significant only in the Lena strain (*p* = 0.018) (Figure 5B). Change in [SWC3+ , SWC9−, SWC8+] granulocytes was not observed in BALF (data not shown).

Without infection, lymphocytes were rarely detected in BALF. The proportion of CD3+ T cells increased up to 18 and 25% from 15 dpi for the Lena- and Finistere-infected pigs, respectively (Figure 5C). CD21+ B cells increased in BALF only at 22 dpi for the Finistere infection (Figure 5D).

### Dynamics of inflammation-related cytokines in BALF and serum

**Figure 6:**
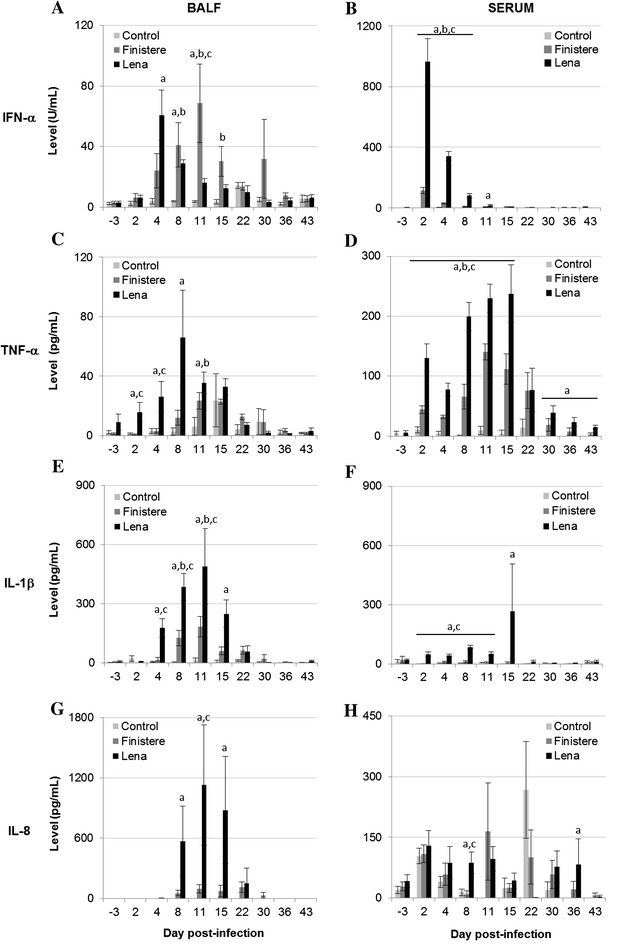
**Pro-inflammatory cytokine profiles in BALF and serum.** Cytokine levels were quantified in BALF (**A**, **C**, **E**, **G**) and sera (**B**, **D**, **F**, **H**) using ELISA for (**A**, **B**) IFN-α, (**C**, **D**) TNF-α, (**E**, **F**) IL-1β and (**G**, **H**) IL-8. All data are reported as the mean (±SE) of results obtained for the pigs in the Control or Finistere groups (*n* = 5 in each group) or the surviving pigs (*n* = 5–8) in the Lena group. “a” indicates a significant difference between the Control and Lena groups, “b” a significant difference between the Control and Finistere groups and “c” a significant difference between the Lena and Finistere groups using a global Kruskal–Wallis test followed by a Holm’s adjusted pairwise Wilcoxon-Mann-Whitney test (*p* < 0.05).

PRRSV infection induced IFN-α production in BALF with an early increase for the Lena infection (maximum mean values of 61 U/mL at 4 dpi for Lena and 69 U/mL at 11 dpi for the Finistere strain) (Figure 6A). In serum, high IFN-α levels were detected after Lena infection (maximum mean value of 964 U/mL at 2 dpi), whereas the Finistere strain simultaneously induced lower IFN-α levels (maximum mean value of 114 U/mL at 2 dpi) (Figure 6B). Similar results were obtained for TNF-α. Compared with the Finistere infection, earlier TNF-α induction was observed in BALF and higher quantities were detected in serum for the Lena infection (Figures 6C and D). Serum levels for IFN-α and TNF-α were correlated with hyperthermia in Lena-infected pigs (r = 0.61 and 0.51, respectively), but not in Finistere-infected pigs. The post-infection modulation of IL-1β level showed a different profile, with higher production induced in BALF than in serum compared with pre-infection levels. In BALF collected from Lena-infected group, the IL-1β level was significantly higher than in Control group from 4 to 15 dpi with maximum mean value of 489 pg/mL at 11 dpi. In BALF collected from the Finistere-infected group, the increase of IL-1β was shortened from 8 to 11 dpi and was weaker than in the Lena-infected group with maximum value of 183 pg/mL at 11 dpi (Figure 6E). In serum, no significant modulation of the IL-1β level was observed for the Finistere infection. The IL-1β level increased slightly in serum from the Lena-infected group (Figure 6F). Likewise, Lena infection led to a high amount of IL-8 in BALF from 8 to 15 dpi, reaching 1129 pg/mL at 11 dpi, whereas no significant levels of IL-8 were detected in BALF for the Finistere infection (Figure 6G). In serum, IL-8 levels were low in all three groups (Figure 6H). BALF IL-1β or IL-8 levels and clinical score were strongly correlated in the Lena group (r = 0.86 and 0.89 respectively), but not in the Finistere group. IL-1β levels were inversely correlated with the viability of BALF cells in the Lena group (r = −0.710), as well as in the Finistere group (r = −0.540).

### Dynamics of cell-mediated immunity-related cytokines in BALF and serum

**Figure 7:**
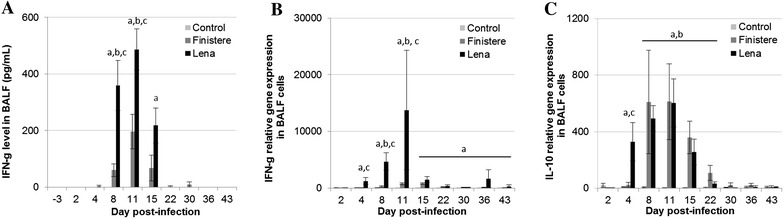
**IFN-γ and IL-10 quantifications in BALF. A** IFN-γ level was quantified in BALF using ELISA. **B** IFN-γ and **C** IL-10 gene expressions were measured in BALF cells by RT-PCR. The presented values are the relative expressions calculated from the cycle threshold (Ct) results on each post-infection day as a function of those obtained the day before the infection as described in “Materials and methods” section. All data are reported as the mean (±SE) of results obtained for the pigs in the Control or Finistere group (*n* = 5 in each group) or the surviving pigs (*n* = 5–8) in Lena group. “a” indicates a significant difference between the Control and Lena groups, “b” a significant difference between the Control and Finistere groups and “c” a significant difference between the Lena and Finistere groups using a global Kruskal–Wallis test followed by a Holm’s adjusted pairwise Wilcoxon-Mann-Whitney test (*p* < 0.05).

Compared with the Finistere-infected group, Lena-infected pigs showed high IFN-γ levels from 8 to 15 dpi in BALF (487 and 196 pg/mL at 11 dpi for the Lena- and Finistere-infected-groups, respectively, *p* = 0.035) (Figure 7A). In serum, IFN-γ was not detected by ELISA for any group.

We further investigated the cellular origin of IFN-γ detected in BALF by studying IFN-γ gene expression in BALF cells by RT-PCR. The results showed an over-expression of IFN-γ after PRRSV infection in cells isolated from BALF compared with the basal expression level in these cells before infection (−3 dpi). PRRSV infection with Lena strain showed a stimulation of IFN-γ gene expression from 4 dpi with maximum value obtained at 11 dpi, the same day as the maximum IFN-γ level in BALF by ELISA (Figure 7B). Compared with the Lena infection, the Finistere infection showed significantly lower IFN-γ gene expression in BALF cells from 4 to 11 dpi (*p* = 0.018 at 4 and 8 dpi; *p* = 0.024 at 11 dpi).

Immunosuppressive cytokine IL-10 was assessed by ELISA, but could not be detected in BALF or serum from any of the pigs. Although the IL-10 protein was not detected by ELISA, expression of IL-10 gene was measured in BALF cells using RT-PCR. Up-regulation of IL-10 gene expression occurred in BALF cells after PRRSV infection without any difference between strains, and only a delayed increase observed for the Finistere strain (increase from 4 dpi for Lena and 8 dpi for the Finistere infection) (Figure 7C).

## Discussion

As previously described, we observed severe respiratory disorders and high fever after infection with the genotype 1.3 Lena strain, but very mild clinical signs with a typical genotype 1.1 strain [7, 17]. Furthermore, despite the absence of any other pathogen, this study showed high mortality in SPF Lena-infected pigs, which confirms its qualification as highly pathogenic. Previous studies have described the death of 4 out of 10 conventional pigs after Lena infection, whereas no mortality occurred in SPF cross-breeds between the Great Yorkshire, Large White type and Dutch Landrace [7, 17]. Susceptibility to PRRSV infection may vary with pig breed, as suggested by Lewis et al. [29], and may explain the high mortality rate we observed in pure Large White SPF pigs. More severe disease symptoms have already been reported for HP-PRRSV infection in Large White pigs compared with either indigenous Chinese or Tibetan breeds [30, 31]. Furthermore, in our study, the mortality observed in SPF pigs could be exacerbated by commensal micro-organisms that became pathogenic in the context of Lena-induced immunosuppression. This second hypothesis is supported by the lesions observed (fibrinous peritonitis) in 2 out of the 3 dead Lena-infected pigs (#4915 and #4917).

We hereby provide the first report of severe and long-lasting anaemia after Lena infection. Using genotype 2 PRRSV strains, Halbur et al. showed that severity of anaemia depends on strain virulence and suggested that the anaemia was probably due to a direct or indirect effect of the virus on erythroid precursor cells in the bone marrow [32]. Similarly, anaemia may have exacerbated the respiratory disorders in Lena-infected pigs by reducing the gas exchanges in the lungs [33].

In this study, we focused on investigating PAMs, which are the primary target cells for PRRSV replication, with relation to clinical score that reflects the virulence of the strain. Comparisons with blood were then carried out to identify lung-specific events. In the literature, reports of the relationships between pathogenicity and PRRSV replication level are contradictory, especially in lung [8, 34]. As in other studies [17, 35–37], our results support the concept that HP strains induce higher viremia, as well as higher viral load in lung than LP strains [17, 35–37]. On the other hand, we demonstrated, as Weesendorp et al. in lung tissues [20], that the Lena strain is cleared faster in BALF than the Finistere strain, suggesting a longer persistence of genotype 1.1 LP-PRRSV strains in the lungs, which may contribute to longer susceptibility to secondary infections of pigs infected by LP strains.

In this study, we showed a faster reduction in viable cells, phagocytic cells and the macrophage population in BALF in the Lena group. We assume that cell death occurs in BALF macrophages (also identified as PAMs), which are phagocytic cells. In vitro, an increase of apoptosis in PAMs has been described after PRRSV infection [38] with extensive severity for the Lena strain [39]. In lung, a higher number of apoptotic cells was observed after infection by the genotype 1.3 HP-PRRSV SU1-Bel strain compared with 1.1 LP-PRRSV strains [40]. Interestingly, in our study, cellular viability in BALF was correlated with viral load, but also with IL-1β levels. As IL-1β is known to induce pyroptosis [41, 42], it suggests that the decrease in macrophages observed in BALF may be due to the direct effect of PRRSV infection, as well as to the effect of pro-apoptotic cytokines such as IL-1β.

In addition to the reduction in phagocytic cell number, resulting from the reduction of macrophage population, phagocytic activity decreased within the remaining phagocytic cells after PRRSV infection with a faster and stronger effect for the Lena strain. Down-regulation of the phagocytic capacity of PRRSV-infected PAMs has previously been shown in vitro; our study presents the first evidence in vivo [23, 43]. Phagocytosis impairment and, consequently, the decrease in the bactericidal activity of macrophages in BALF may contribute to lung damage by compromising homeostasis and favouring the development of secondary bacterial infections. Thereafter, renewal of macrophages in BALF may be triggered by an influx of monocytes from blood at 8–11 dpi (with more efficient recruitment in Lena infection), which may promote virus replication and exacerbate the production of inflammatory cytokines. However, recent studies have shown that some macrophages can originate and renew in adult tissues independently from hematopoietic stem cells, but derived instead from yolk sac foetal monocytes [44, 45].

After having investigated the macrophage population and their activity, we then extended our study to the cytokines present in BALF. In blood, as previously described [16, 17], PRRSV infection with HP strains enhanced pro-inflammatory response compared with LP strains [16, 17]. We showed that IFN-α or TNF-α response in blood correlates with the hyperthermia observed after Lena infection. Our results support the hypothesis that the stronger systemic inflammatory response induced after HP strains infection is responsible for the high fever that specifically occurs with these strains. In contrast, in BALF, the major difference between strains was a lag in onset of the IFN-α or TNF-α maximum levels in the Finistere strain infection. These results suggest weak stimulation of Lena strain in the lung and indicate that the high levels of IFN-α and TNF-α detected in the blood are probably produced in other tissues. This low induction of IFN-α or TNF-α in lung is well described for PRRSV infection and reflects the PRRSV evasion from the host innate response [46–49]. Comparing different respiratory pathogens, levels of pro-inflammatory cytokines in lung have been linked to the severity of the pulmonary lesions [15]. There were no significant differences in IFN-α or TNF-α maximum levels between the Lena and Finistere strains, suggesting that IFN-α and TNF-α are not the main inflammatory cytokines involved in lung damage.

Interestingly, the low modulations detected for IFN-α and TNF-α levels contrast with exacerbated levels of IL-1β and IL-8 measured in BALF. Even if no effect of the repeated bronchoalveolar lavages was noticed in the Control group, we cannot completely exclude an additional effect of BAL in inducing inflammation in the lungs of PRRS infected animals. However, up-regulation of IL-1β gene expression in lung after Lena-infection has previously been reported by Weesendorp el al. [20]. Amarilla et al. recently showed a correlation between IL-1α and the intensity of clinical signs and lung lesions in pigs infected with the HP SU1-bel PRRSV strain [50]. Our results confirm and provide further evidence for a relationship between IL-1β levels in lung and the severity of the disease. Furthermore, our study presents the first evidence that IL-8 is over-produced after infection with an HP strain, but not LP strains, within the lungs. Previous studies on genotype 2 strains have reported the up-regulation of IL-8 gene expression in PAMs isolated from infected pigs using a Chinese HP strain at 14 dpi [51] or in in vitro-infected PAMs using the VR-2332 strain [52]. Our results demonstrate a dramatic lung-specific IL-8 protein production after infection with a HP strain compared with a LP strain, suggesting an essential role of this cytokine in lung pathogenesis. IL-8 is a pro-inflammatory cytokine mainly secreted by monocytes and endothelial cells, which chemoattract neutrophils. For many respiratory pathogens such as the porcine influenza virus, *A. pleuropneumoniae* or *M. hyopneumoniae*, neutrophil lung infiltration causes pneumonia and is related to the severity of disease [53–55]. During PRRSV infection, neutrophil lung invasion is frequently described for HP strains [18, 20, 56]. The coincidence in IL-8 and monocyte detection in BALF suggests that IL-8 may originate from recruited monocytes rather than PAMs in the Lena infection. Likewise, the high level of IL-1β induced during the Lena infection may result from additional production of IL-1β by recruited monocytes.

In viral infections, adaptive immunity can be influenced by the innate response toward the interaction of IFN-α with dendritic cells, promoting the development of T cell-mediated IFN-γ responses to enhance anti-viral immune defences, or directly towards IFN-γ produced by NK cells [57]. Although low IFN-α levels were detected in BALF for both PRRSV strains, earlier IFN-α induction was observed in the Lena infection, which may promote a stronger IFN-γ response resulting in a faster virus clearance in BALF than for the Finistere strain. The IFN-γ levels detected in BALF could also originate from NK cells as higher proportions of NK cells and CD8+ T cells were detected at 15 dpi in BALF from PRRSV-infected pigs (data not shown) and we could hypothesize that the increase of NK cells occurred earlier in Lena- than in Finistere-infected pigs. IFN-γ has effectively been shown to inhibit viral replication in macrophages and MARC-145 and is associated with clinical protection against PRRSV infection [58–60]. In our study, the high levels of IFN-γ measured in BALF from Lena-infected pigs corroborate previous results that show a similar IFN-γ response after infection with the HP genotype 1.3 SU1-bel strain [8], but contrast with the results indicating lower ELISPOT IFN-γ BALF cell response for the Lena strain than for the LP Lelystad strain at 35 dpi [20]. There was no difference in the immunosuppressive IL-10 gene expression in BALF cells between strains, supporting a balance toward a stronger cell-mediated immune response and/or a stronger innate NK cell response after Lena infection than after Finistere infection.

In conclusion, our data indicate that the HP Lena strain markedly alters the bronchoalveolar cellular populations and cytokines in conjunction with a high virus load and severe respiratory clinical signs. The PAM population reduced rapidly, coinciding with lower cellular viability and phagocytic activity that probably reduce the bactericidal activity of macrophages and increase susceptibility to secondary infections. The IL-1β and IL-8 inflammatory response was stronger in BALF for the Lena infection, and may promote lung damage and limit gas exchanges. In later stages of the PRRSV infection, faster viral clearance in BALF from Lena-infected pigs may result from a more effective IFN-γ T cell and/or NK cell response. Improving the knowledge on PRRSV pathogenesis and on local immune responses induced post-vaccination can lead to the development of more efficient vaccine strategies to control the disease.

## Additional file

**Additional file 1.** **BALF cell count.** Cell concentrations in BALF were counted using a haemocytometer with Trypan blue staining. All data are reported as the mean (±SE) of results obtained for the pigs in the Control or Finistere groups (*n* = 5 in each group) or the surviving pigs (*n* = 5–8) in the Lena group. * at 22 dpi, the cell concentrations for Control and Finistere group were underestimated due to the high level of mucus in the BALF for the animals of these groups.

## Abbreviations

PRRSV: porcine reproductive and respiratory syndrome virus
SPF: specific-pathogen-free
PAMs: pulmonary alveolar macrophages
HP: highly pathogenic
LP: low pathogenic
BALF: bronchoalveolar lavage fluid
IFN: interferon
IL: interleukin
TNF: tumour necrosis factor
MFI: median fluorescence intensity
CPD: cytopathogenic dose
ADWG: average daily weight gain

## References

[1] Neumann EJ, Kliebenstein JB, Johnson CD, Mabry JW, Bush EJ, Seitzinger AH, Green AL, Zimmerman JJ (2005). Assessment of the economic impact of porcine reproductive and respiratory syndrome on swine production in the United States. J Am Vet Med Assoc.

[2] Done SH, Paton DJ (1995). Porcine reproductive and respiratory syndrome: clinical disease, pathology and immunosuppression. Vet Rec.

[3] Conzelmann KK, Visser N, Van Woensel P, Thiel HJ (1993). Molecular characterization of porcine reproductive and respiratory syndrome virus, a member of the arterivirus group. Virology.

[4] Lunney JK, Benfield DA, Rowland RR (2010). Porcine reproductive and respiratory syndrome virus: an update on an emerging and re-emerging viral disease of swine. Virus Res.

[5] Kappes MA, Faaberg KS (2015). PRRSV structure, replication and recombination: origin of phenotype and genotype diversity. Virology.

[6] Stadejek T, Oleksiewicz MB, Potapchuk D, Podgorska K (2006). Porcine reproductive and respiratory syndrome virus strains of exceptional diversity in eastern Europe support the definition of new genetic subtypes. J Gen Virol.

[7] Karniychuk UU, Geldhof M, Vanhee M, Van Doorsselaere J, Saveleva TA, Nauwynck HJ (2010). Pathogenesis and antigenic characterization of a new East European subtype 3 porcine reproductive and respiratory syndrome virus isolate. BMC Vet Res.

[8] Morgan SB, Graham SP, Salguero FJ, Sanchez Cordon PJ, Mokhtar H, Rebel JM, Weesendorp E, Bodman-Smith KB, Steinbach F, Frossard JP (2013). Increased pathogenicity of European porcine reproductive and respiratory syndrome virus is associated with enhanced adaptive responses and viral clearance. Vet Microbiol.

[9] Chand RJ, Trible BR, Rowland RR (2012). Pathogenesis of porcine reproductive and respiratory syndrome virus. Curr Opin Virol.

[10] Gomez-Laguna J, Salguero FJ, Pallares FJ, Carrasco L (2013). Immunopathogenesis of porcine reproductive and respiratory syndrome in the respiratory tract of pigs. Vet J.

[11] Morales-Nebreda L, Misharin AV, Perlman H, Budinger GR (2015). The heterogeneity of lung macrophages in the susceptibility to disease. Eur Respir Rev.

[12] Werner JL, Steele C (2014). Innate receptors and cellular defense against pulmonary infections. J Immunol.

[13] Divangahi M, King IL, Pernet E (2015). Alveolar macrophages and type I IFN in airway homeostasis and immunity. Trends Immunol.

[14] Mair KH, Sedlak C, Kaser T, Pasternak A, Levast B, Gerner W, Saalmuller A, Summerfield A, Gerdts V, Wilson HL, Meurens F (2014). The porcine innate immune system: an update. Dev Comp Immunol.

[15] Van Reeth K, Labarque G, Nauwynck H, Pensaert M (1999). Differential production of proinflammatory cytokines in the pig lung during different respiratory virus infections: correlations with pathogenicity. Res Vet Sci.

[16] Liu Y, Shi W, Zhou E, Wang S, Hu S, Cai X, Rong F, Wu J, Xu M, Xu M, Li L (2010). Dynamic changes in inflammatory cytokines in pigs infected with highly pathogenic porcine reproductive and respiratory syndrome virus. Clin Vaccine Immunol.

[17] Weesendorp E, Morgan S, Stockhofe-Zurwieden N, Popma-De Graaf DJ, Graham SP, Rebel JM (2013). Comparative analysis of immune responses following experimental infection of pigs with European porcine reproductive and respiratory syndrome virus strains of differing virulence. Vet Microbiol.

[18] Han D, Hu Y, Li L, Tian H, Chen Z, Wang L, Ma H, Yang H, Teng K (2014). Highly pathogenic porcine reproductive and respiratory syndrome virus infection results in acute lung injury of the infected pigs. Vet Microbiol.

[19] Lunney JK, Fang Y, Ladinig A, Chen N, Li Y, Rowland B, Renukaradhya GJ (2016). Porcine reproductive and respiratory syndrome virus (PRRSV): pathogenesis and interaction with the immune system. Annu Rev Anim Biosci.

[20] Weesendorp E, Rebel JM, Popma-De Graaf DJ, Fijten HP, Stockhofe-Zurwieden N (2014). Lung pathogenicity of European genotype 3 strain porcine reproductive and respiratory syndrome virus (PRRSV) differs from that of subtype 1 strains. Vet Microbiol.

[21] Rose N, Renson P, Andraud M, Paboeuf F, Le Potier MF, Bourry O (2015). Porcine reproductive and respiratory syndrome virus (PRRSv) modified-live vaccine reduces virus transmission in experimental conditions. Vaccine.

[22] Piriou-Guzylack L, Salmon H (2008). Membrane markers of the immune cells in swine: an update. Vet Res.

[23] De Baere MI, Van Gorp H, Nauwynck HJ, Delputte PL (2011). Antibody binding to porcine sialoadhesin reduces phagocytic capacity without affecting other macrophage effector functions. Cell Immunol.

[24] Nielsen J, Botner A, Tingstedt JE, Aasted B, Johnsen CK, Riber U, Lind P (2003). In utero infection with porcine reproductive and respiratory syndrome virus modulates leukocyte subpopulations in peripheral blood and bronchoalveolar fluid of surviving piglets. Vet Immunol Immunopathol.

[25] Jamin A, Gorin S, Cariolet R, Le Potier MF, Kuntz-Simon G (2008). Classical swine fever virus induces activation of plasmacytoid and conventional dendritic cells in tonsil, blood, and spleen of infected pigs. Vet Res.

[26] Petrov A, Beer M, Blome S (2014). Development and validation of a harmonized TaqMan-based triplex real-time RT-PCR protocol for the quantitative detection of normalized gene expression profiles of seven porcine cytokines. PLoS One.

[27] Royaee AR, Husmann RJ, Dawson HD, Calzada-Nova G, Schnitzlein WM, Zuckermann FA, Lunney JK (2004). Deciphering the involvement of innate immune factors in the development of the host response to PRRSV vaccination. Vet Immunol Immunopathol.

[28] Livak KJ, Schmittgen TD (2001). Analysis of relative gene expression data using real-time quantitative PCR and the 2^−∆∆C(T)^ method. Methods.

[29] Lewis CR, Ait-Ali T, Clapperton M, Archibald AL, Bishop S (2007). Genetic perspectives on host responses to porcine reproductive and respiratory syndrome (PRRS). Viral Immunol.

[30] Liang W, Li Z, Wang P, Fan P, Zhang Y, Zhang Q, Wang Y, Xu X, Liu B (2016). Differences of immune responses between Tongcheng (Chinese local breed) and Large White pigs after artificial infection with highly pathogenic porcine reproductive and respiratory syndrome virus. Virus Res.

[31] Kang R, Ji G, Yang X, Lv X, Zhang Y, Ge M, Pan Y, Li Q, Wang H, Zeng F (2016). Investigation on host susceptibility of Tibetan pig to infection of porcine reproductive and respiratory syndrome virus through viral challenge study. Vet Microbiol.

[32] Halbur RG, Pallares FJ, Rathje JA, Evans R, Hagemoser WA, Paul PS, Meng XJ (2002). Effects of different us isolates of porcine reproductive and respiratory syndrome virus (PRRSV) on blood and bone marrow parameters of experimentally infected pigs. Vet Rec.

[33] Guo J, Zheng C, Xiao Q, Gong S, Zhao Q, Wang L, He J, Yang W, Shi X, Sun X, Liu J (2015). Impact of anaemia on lung function and exercise capacity in patients with stable severe chronic obstructive pulmonary disease. BMJ Open.

[34] Weesendorp E, Stockhofe-Zurwieden N, Nauwynck HJ, Popma-De Graaf DJ, Rebel JM (2016). Characterization of immune responses following homologous reinfection of pigs with European subtype 1 and 3 porcine reproductive and respiratory syndrome virus strains that differ in virulence. Vet Microbiol.

[35] Han Z, Liu Y, Wang G, He Y, Hu S, Li Y, Shi W, Wu J, Wang S, Liu H, Cai X (2015). Comparative analysis of immune responses in pigs to high and low pathogenic porcine reproductive and respiratory syndrome viruses isolated in China. Transbound Emerg Dis.

[36] Hu SP, Zhang Z, Liu YG, Tian ZJ, Wu DL, Cai XH, He XJ (2013). Pathogenicity and distribution of highly pathogenic porcine reproductive and respiratory syndrome virus in pigs. Transbound Emerg Dis.

[37] Johnson W, Roof M, Vaughn E, Christopher-Hennings J, Johnson CR, Murtaugh MP (2004). Pathogenic and humoral immune responses to porcine reproductive and respiratory syndrome virus (PRRSV) are related to viral load in acute infection. Vet Immunol Immunopathol.

[38] Costers S, Lefebvre DJ, Delputte PL, Nauwynck HJ (2008). Porcine reproductive and respiratory syndrome virus modulates apoptosis during replication in alveolar macrophages. Arch Virol.

[39] Weesendorp E, Stockhofe-Zurwieden N, Popma-De Graaf DJ, Fijten H, Rebel JM (2013). Phenotypic modulation and cytokine profiles of antigen presenting cells by European subtype 1 and 3 porcine reproductive and respiratory syndrome virus strains in vitro and in vivo. Vet Microbiol.

[40] Morgan SB, Frossard JP, Pallares FJ, Gough J, Stadejek T, Graham SP, Steinbach F, Drew TW, Salguero FJ (2016). Pathology and virus distribution in the lung and lymphoid tissues of pigs experimentally inoculated with three distinct type 1 PRRS virus isolates of varying pathogenicity. Transbound Emerg Dis.

[41] Chow SH, Deo P, Naderer T (2016). Macrophage cell death in microbial infections. Cell Microbiol.

[42] Miao EA, Rajan JV, Aderem A (2011). Caspase-1-induced pyroptotic cell death. Immunol Rev.

[43] Tsai YC, Chang HW, Jeng CR, Lin TL, Lin CM, Wan CH, Pang VF (2012). The effect of infection order of porcine circovirus type 2 and porcine reproductive and respiratory syndrome virus on dually infected swine alveolar macrophages. BMC Vet Res.

[44] Gomez Perdiguero E, Klapproth K, Schulz C, Busch K, Azzoni E, Crozet L, Garner H, Trouillet C, de Bruijn MF, Geissmann F, Rodewald HR (2015). Tissue-resident macrophages originate from yolk-sac-derived erythro-myeloid progenitors. Nature.

[45] Schulz C, Gomez Perdiguero E, Chorro L, Szabo-Rogers H, Cagnard N, Kierdorf K, Prinz M, Wu B, Jacobsen SE, Pollard JW, Frampton J, Liu KJ, Geissmann F (2012). A lineage of myeloid cells independent of Myb and hematopoietic stem cells. Science.

[46] Albina E, Carrat C, Charley B (1998). Interferon-alpha response to swine arterivirus (PoAV), the porcine reproductive and respiratory syndrome virus. J Interferon Cytokine Res.

[47] van Reeth K, Nauwynck H (2000). Proinflammatory cytokines and viral respiratory disease in pigs. Vet Res.

[48] Sun Y, Han M, Kim C, Calvert JG, Yoo D (2012). Interplay between interferon-mediated innate immunity and porcine reproductive and respiratory syndrome virus. Viruses.

[49] Garcia-Nicolas O, Quereda JJ, Gomez-Laguna J, Salguero FJ, Carrasco L, Ramis G, Pallares FJ (2014). Cytokines transcript levels in lung and lymphoid organs during genotype 1 porcine reproductive and respiratory syndrome virus (PRRSV) infection. Vet Immunol Immunopathol.

[50] Amarilla SP, Gomez-Laguna J, Carrasco L, Rodriguez-Gomez IM, Caridad YOJM, Morgan SB, Graham SP, Frossard JP, Drew TW, Salguero FJ (2015). A comparative study of the local cytokine response in the lungs of pigs experimentally infected with different PRRSV-1 strains: upregulation of IL-1alpha in highly pathogenic strain induced lesions. Vet Immunol Immunopathol.

[51] Xiao Y, An TQ, Tian ZJ, Wei TC, Jiang YF, Peng JM, Zhou YJ, Cai XH, Tong GZ (2015). The gene expression profile of porcine alveolar macrophages infected with a highly pathogenic porcine reproductive and respiratory syndrome virus indicates overstimulation of the innate immune system by the virus. Arch Virol.

[52] Miller LC, Neill JD, Harhay GP, Lager KM, Laegreid WW, Kehrli ME (2010). In-depth global analysis of transcript abundance levels in porcine alveolar macrophages following infection with porcine reproductive and respiratory syndrome virus. Adv Virol.

[53] Van Reeth K, Nauwynck H, Pensaert M (1998). Bronchoalveolar interferon-alpha, tumor necrosis factor-alpha, interleukin-1, and inflammation during acute influenza in pigs: a possible model for humans?. J Infect Dis.

[54] Morrison DF, Foss DL, Murtaugh MP (2000). Interleukin-10 gene therapy-mediated amelioration of bacterial pneumonia. Infect Immun.

[55] Meyns T, Maes D, Calus D, Ribbens S, Dewulf J, Chiers K, de Kruif A, Cox E, Decostere A, Haesebrouck F (2007). Interactions of highly and low virulent *Mycoplasma hyopneumoniae* isolates with the respiratory tract of pigs. Vet Microbiol.

[56] Liu J, Hou M, Yan M, Lu X, Gu W, Zhang S, Gao J, Liu B, Wu X, Liu G (2015). ICAM-1-dependent and ICAM-1-independent neutrophil lung infiltration by porcine reproductive and respiratory syndrome virus infection. Am J Physiol Lung Cell Mol Physiol.

[57] Loving CL, Osorio FA, Murtaugh MP, Zuckermann FA (2015). Innate and adaptive immunity against porcine reproductive and respiratory syndrome virus. Vet Immunol Immunopathol.

[58] Bautista EM, Molitor TW (1999). IFN gamma inhibits porcine reproductive and respiratory syndrome virus replication in macrophages. Arch Virol.

[59] Rowland RR, Robinson B, Stefanick J, Kim TS, Guanghua L, Lawson SR, Benfield DA (2001). Inhibition of porcine reproductive and respiratory syndrome virus by interferon-gamma and recovery of virus replication with 2-aminopurine. Arch Virol.

[60] Lowe JE, Husmann R, Firkins LD, Zuckermann FA, Goldberg TL (2005). Correlation of cell-mediated immunity against porcine reproductive and respiratory syndrome virus with protection against reproductive failure in sows during outbreaks of porcine reproductive and respiratory syndrome in commercial herds. J Am Vet Med Assoc.

