# Supplementary figures and images for: Dynamic changes in bronchoalveolar macrophages and cytokines during infection of pigs with a highly or low pathogenic genotype 1 PRRSV strain
Source: Vet Res. 2017 Feb 27;48:15. doi: 10.1186/s13567-017-0420-y (PMC5327547; doi:10.1186/s13567-017-0420-y)

## Slide 1
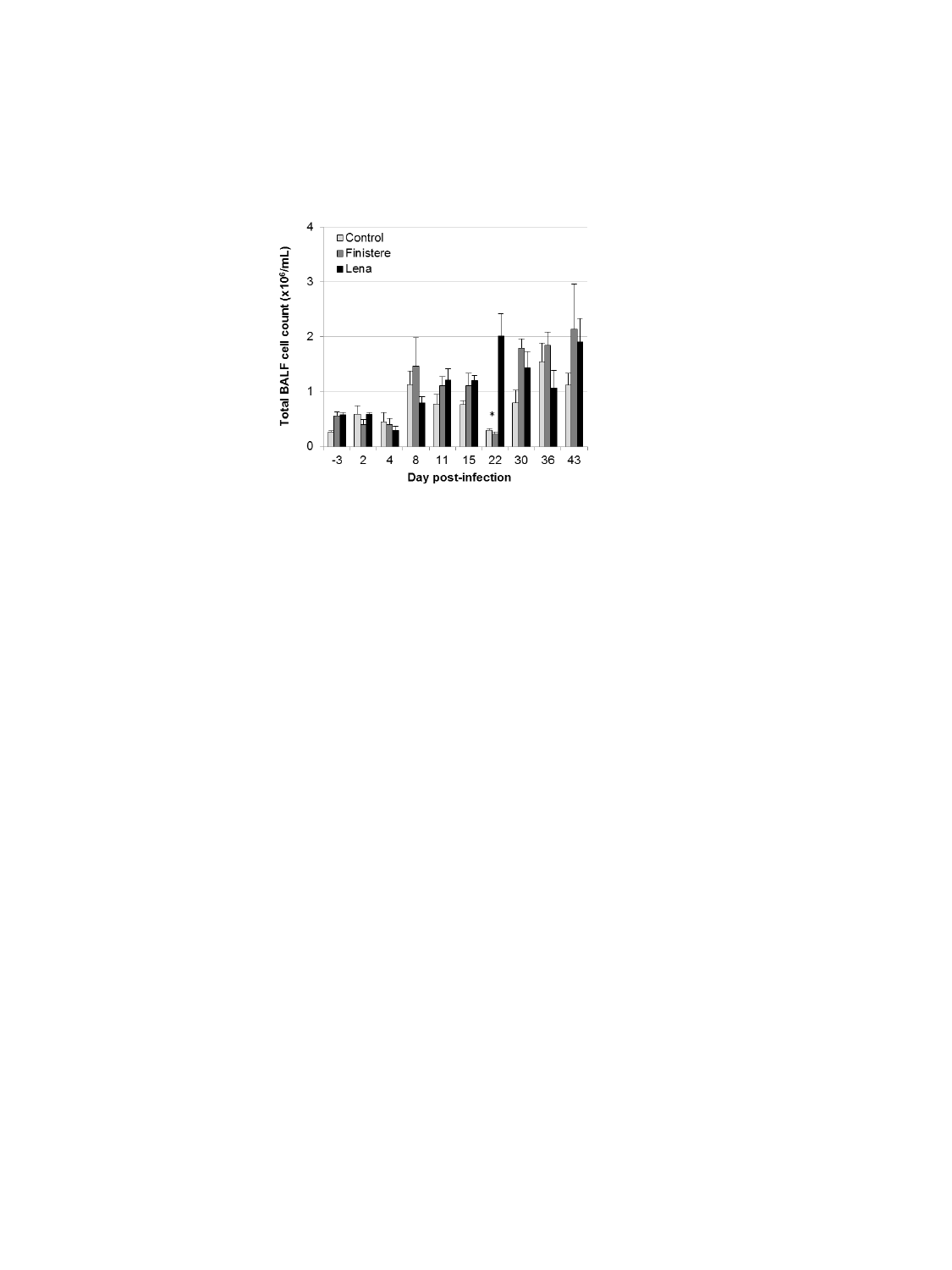

Supplement: Supplementary file 1 — Additional file 1. BALF cell count. Cell concentrations in BALF were counted using a haemocytometer with Trypan blue staining. All data are reported as the mean (±SE) of results obtained for the pigs in the Control or Finistere groups (n = 5 in each group) or the surviving pigs (n = 5–8) in the Lena group. * at 22 dpi, the cell concentrations for Control and Finistere group were underestimated due to the high level of mucus in the BALF for the animals of these groups. [file 13567_2017_420_MOESM1_ESM.pptx]
